# Corrigendum to “Efficacy and safety of bridging intravenous thrombolysis before mechanical thrombectomy: A systematic review and meta-analysis”

**DOI:** 10.1177/15910199251401478

**Published:** 2025-12-08

Hashmi TM, Ahmed M, Ashraf H, et al. Efficacy and safety of bridging intravenous thrombolysis before mechanical thrombectomy: A systematic review and meta-analysis. *Interv Neuroradiol* 2025. doi:10.1177/15910199251368728

In the above published article, the article title has been corrected, and the updated title reads as below:

Efficacy and safety of bridging intravenous thrombolysis before mechanical thrombectomy in acute ischemic stroke: A systematic review and meta-analysis

The online version of this article has been updated.

